# Chromatin Dynamics in the Regulation of *CFTR* Expression

**DOI:** 10.3390/genes6030543

**Published:** 2015-07-13

**Authors:** Nehal Gosalia, Ann Harris

**Affiliations:** 1 Human Molecular Genetics Program, Lurie Children’s Research Center, Chicago, IL 60614, USA; E-Mail: nehalgosalia2014@u.northwestern.edu; 2 Department of Pediatrics, Northwestern University Feinberg School of Medicine, Chicago, IL 60611, USA

**Keywords:** *CFTR*, *cis*-regulatory elements, higher order chromatin organization

## Abstract

The contribution of chromatin dynamics to the regulation of human disease-associated loci such as the cystic fibrosis transmembrane conductance regulator (*CFTR*) gene has been the focus of intensive experimentation for many years. Recent technological advances in the analysis of transcriptional mechanisms across the entire human genome have greatly facilitated these studies. In this review we describe the complex machinery of tissue-specific regulation of *CFTR* expression, and put earlier observations in context by incorporating them into datasets generated by the most recent genomics methods. Though the gene promoter is required for *CFTR* expression, cell-type specific regulatory elements are located elsewhere in the gene and in flanking intergenic regions. Probably within its own topological domain established by the architectural proteins CTCF and cohesin, the *CFTR* locus utilizes chromatin dynamics to remodel nucleosomes, recruit cell-selective transcription factors, and activate intronic enhancers. These *cis*-acting elements are then brought to the gene promoter by chromatin looping mechanisms, which establish long-range interactions across the locus. Despite its complexity, the *CFTR* locus provides a paradigm for elucidating the critical role of chromatin dynamics in the transcription of individual human genes.

## 1. Introduction

One of the most intensively studied genes associated with human disease is the cystic fibrosis transmembrane conductance regulator (*CFTR*) gene, which encompasses 189 kb at chromosome 7q31.2. It encodes a 1480 amino acid protein, which primarily functions as a cAMP-dependent chloride ion channel [1,2,3] belonging to the ATP-binding cassette (ABC) transporter superfamily. Mutations in *CFTR* cause the common, autosomal recessive disease cystic fibrosis (CF) [4,5,6], which is characterized by high salt levels in the sweat and the accumulation of thick, sticky mucus, which is associated with inflammation, tissue fibrosis, and impaired lung, intestinal, and pancreatic function. The *CFTR* gene shows tight, tissue-specific regulation of expression. The CFTR protein is abundant in the specialized epithelial cells of tissues associated with CF pathology such as the intestine, pancreas, sweat gland, and genital ducts, though levels are much lower in the respiratory epithelium [7,8,9,10]. It is also found in some other organ and cell sites. Within the intestinal epithelium there is a strong gradient of expression along the crypt (high) to the villous (low) axis and from the proximal to distal intestine [8]. *CFTR* is expressed from early in gestation [11] and shows temporal regulation in the lung, with the fetal lung having higher levels of transcript than adult tissue [12,13,14].

This complex pattern of coordinated tissue-specific and temporal regulation led to intensive investigation of the mechanisms controlling *CFTR* gene expression. These studies, which were among the first to describe critical regulatory elements in non-coding regions of the genome [15], provided a paradigm for understanding gene regulation genome-wide. The subsequent dramatic technological and conceptual advances made by the Encyclopedia of DNA elements (ENCODE) consortium [16] provided new tools to further delineate the mechanisms of *CFTR* regulation, particularly in differentiated primary epithelial cell types. Early work on the transcriptional regulation of *CFTR* focused on the promoter, which was classified as a “house-keeping” gene-like promoter. Analysis of a ~4 kb region upstream of the first exon showed a high GC content, no TATA box, and multiple transcription start sites (TSS) [17,18,19]. Utilization of different TSS may be involved in both developmental and tissue-specific control of the gene, for example, with different start sites used in the fetal and adult lung [20]. Moreover, *CFTR* gene promoter activity may be regulated by the recruitment of alternative 5' exons and local RNA secondary structure [21,22,23]. Initial studies also showed that the promoter contained binding sites for the transcription factors Sp1 and AP-1 [17], a cAMP response element (CRE), a CCAAT box [24,25,26], and a NF-κB binding site [27,28]. However, these analyses did not identify the mechanisms by which tissue-specific *CFTR* expression is achieved. The lack of cell-type specific control elements in the promoter (defined here as ~2kb 5' to the translational start site) and the large size of the gene, with multiple long introns, led to the hypothesis that critical *cis*-regulatory elements might exist within the gene body itself or in distal upstream or downstream regions.

## 2. Identification of *cis*-Regulatory Elements in the *CFTR* Locus

**Figure 1:**
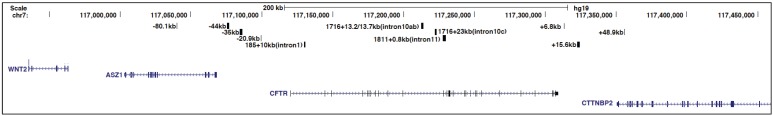
Schematic to show to the genomic location of the cystic fibrosis transmembrane conductance regulator (*CFTR*) locus and some of its key *cis*-regulatory elements discussed in this review. From the University of California, Santa Cruz (UCSC) genome browser.

To identify open chromatin regions and associated regulatory elements; within and flanking the *CFTR* gene, DNase I hypersensitive site (DHS) mapping was performed. The first studies used Southern blotting with gene specific probes, but subsequent methodological advances enabled the whole *CFTR* locus to be evaluated by DNase-chip [29] and later DNase-seq [30]. Nucleosomes can prevent transcription factors from accessing their binding sites within *cis*-regulatory elements, therefore nucleosome-depleted regions, which are hypersensitive to digestion by DNase I, represent potentially active regulatory elements [31]. Initial analyses on *CFTR* by Southern blotting revealed DHS at −79.5 kb and −20.9 kb from the translational start site of *CFTR* [32], within several introns of the gene [15,33], and in the 3' intergenic region [34] (Figure 1). The intronic sites, particularly those within introns 1, 10, 17a, and 20, showed some cell-type restriction, suggesting a role in tissue-specific regulation of *CFTR* expression. Analyses of these elements, primarily using *in vitro* DNase I footprinting, electromobility shift assays (EMSAs), and luciferase reporter gene assays, showed that they bound various transcription factors and were likely essential for *CFTR* expression [15,34,35,36,37,38]. Moreover, the functional importance of these intronic sites was shown by deletion of the DHS in intron 1, which was identified as the first *CFTR* intronic *cis*-element [15], from a yeast artificial chromosome (YAC) encompassing the *CFTR* locus. Loss of this region from the YAC-encoded *CFTR* gene reduced its expression in transgenic mouse intestine by ~60% [39]. However, this deletion had no effect on *CFTR* expression from the YAC in transgenic mouse lung tissue, providing more direct evidence for the importance of this intronic element in tissue-specific regulation of *CFTR* expression. Further characterization of the intestinal-specific DHS in intron 1 showed that it contained hepatocyte nuclear factor 1 homebox A (HNF1α) binding sites and depletion of HNF1α caused a decrease in *CFTR* mRNA levels [40]. Subsequently, the importance of HNF1 binding at this site was confirmed by chromatin immunoprecipitation (ChIP) [41].

## 3. Characterization of Enhancers at the *CFTR* Locus

The realization and more general acceptance in the late 1990s that far from being “junk” DNA, introns contained many of the critical regulatory elements coordinating gene expression across the genome validated the earlier, laborious work on the *CFTR* locus. Genome-wide analyses using new methods developed by the ENCODE consortium revealed many functions for the intronic and intergenic sequences of the genome that act as *cis*-regulatory elements [42]. One functional class that has received much attention is enhancers, which are often located distal to the genes they regulate, utilize chromatin remodelers, and bind to transcriptional activators or co-activators to initiate gene expression. Enhancers frequently show tissue-specificity and are readily identified since they are depleted of nucleosomes, constitute open chromatin regions, and carry specific histone modifications [43].

In addition to the enhancer in the *CFTR* intron 1 DHS described above and a weak enhancer in intron 20 [35], DNase-chip identified several novel DHS in cells expressing different levels of *CFTR* mRNA [44] (Figure 1). Fibroblasts, which do not express *CFTR* lacked intronic DHS, while Caco2 (colorectal adenocarcinoma) cells expressing abundant *CFTR* showed prominent DHS within introns 10 and 11. The novel site in intron 11 was later shown to encompass a very strong enhancer [44] that appears critical for the high levels of *CFTR* expression in the intestinal epithelium. The DNase-chip analysis also showed cell-type selectivity of the DHS at the *CFTR* locus, since sites that were evident in *CFTR*-expressing airway cells were absent in intestinal cells and vice-versa [44]. Many of the intronic DHS in primary cells have not yet been studied intensively and may contribute further insights into the cell- specific fine-tuning of the locus.

The presence or absence of DHS may be regulated through nucleosome positioning and remodeling [45]. Using a new method of nucleosome mapping, based on hybridization of purified nucleosomes to a bacterial artificial chromosome (BAC) encompassing the whole *CFTR* locus, we mapped nucleosomes in several cell types [46]. The intestinal-selective enhancer at the intron 1 DHS was nucleosome-depleted only in Caco2 cells and the HNF1 binding site, which drives this enhancer element, was occluded by nucleosomes in *CFTR*-expressing airway cells and in skin fibroblasts. Similar nucleosome-depleted sequences were observed at another intestinal-specific enhancer in intron 11 and an airway-selective DHS at −35 kb upstream of the translational start site [46]. However, certain ubiquitous DHS such as those at −20.9 kb 5' of the translational start site and +6.8 kb downstream of the last coding base showed a nucleosome-depleted core region across multiple cell types, suggesting occupancy of a common factor.

Enhancer sequences are also associated with specific histone modifications and occupancy by the factors that establish those marks. For example, active enhancers are often associated with acetylation at lysine 27 of histone H3 (H3K27ac) [47,48,49], which is established by p300. ChIP for p300 across the *CFTR* locus in Caco2 cells showed enrichment of the factor at multiple DHS identified by DNase-chip in these cells [44]. Enhancers are also often marked by mono- or di-methylation on lysine 4 of histone H3 (H3K4me1/me2) [47,49]. H3K4me1 was previously shown to be enriched at regions corresponding to cell-type specific DHS and enhancer elements within the *CFTR* locus [50].

## 4. Identification of Transcription Factors Regulating *CFTR*

Although the hallmark features discussed above help to identify putative enhancers, the most important characteristic of these regions is their ability to recruit transcription factors and co-activators that promote gene expression. Studies on post-confluent Caco2 cells, a model of intestinal *CFTR* regulation, identified a network of relevant transcription factors interacting with *cis*-regulatory elements at the locus. These factors included, HNF1, forkhead box protein A (FOXA), and caudal type homeobox 2 (CDX2) [40,44,51]. In Caco2 cells HNF1α was enriched at the intron 1 and 11 enhancers, and at lower levels at a DHS in intron 10 [44]. Subsequently, using *in silico* predictions, *in vitro* DNase I footprinting, EMSAs, and ChIP, the pioneer transcription factors, FOXA1 and FOXA2, were shown to occupy DHS elements in introns 10 and 11, while CDX2 was found at multiple intergenic and intronic DHS across the *CFTR* locus [51]. siRNA-mediated depletion of CDX2 or both FOXA1 and FOXA2 together led to 25% and 40% reductions in *CFTR* mRNA levels, respectively [51,52]. Binding of HNF1 and CDX2 was shown to be dependent on the presence of FOXA1/A2 [52] as was observed for other FOXA1/A2 co-factors in genome-wide studies [53,54]. Interestingly, depletion of the FOXA factors altered enhancer histone modification profiles (H3K27ac and H3K4me2) at specific sites across the *CFTR* locus and led to a decrease in hypersensitivity of a DHS in intron 10 [52]. These data suggest that the transcription factors involved in intestinal regulation of *CFTR* function in a coordinated network, in which the FOXA proteins have a pivotal role.

The DHS and nucleosome positioning data suggest that *CFTR* expression is regulated by different mechanisms in airway and intestinal cells. This is achieved both by utilization of airway-selective *cis*-regulatory elements and by recruitment of distinct transcription factors [44,46]. Analysis of *CFTR* regulation in airway cells revealed DHS at −44 kb and −35 kb upstream of the locus, and also in introns 18, 19, and 23 [55] (Figure 1). The DHS at −35 kb binds to the interferon regulatory factors 1 and 2 (IRF1/2) and nuclear factor Y (NF-Y) [56]. Constitutive occupancy of IRF2 at this site represses *CFTR* expression, but following an interferon-mediated increase in intracellular IRF1 levels, IRF2 is replaced by IRF1, which activates gene expression. NF-Y has an important role in recruiting histone-modifying enzymes to this site, where it interacts with the transcriptional repressor SIN3A and the SET domain containing lysine methyl-transferase 7 (SETD7) [56]. The DHS −35 kb element is functionally linked to regulatory sequences within DHS −44 kb for modulating *CFTR* expression in response to environmental stresses in the airway. DHS −44 kb contains an antioxidant response element (ARE) and is usually bound by the repressors BTB and CNC homology 1, basic leucine zipper transcription factor 1 (Bach1) and v-maf avian musculoaponeurotic fibrosarcoma oncogene homolog K (MafK) [57]. However, upon antioxidant treatment, Bach1/MafK heterodimers are replaced by the transcriptional activator Nrf2, thus enhancing *CFTR* expression. Nucleosome occupancy profiles combined with *in silico* predictions also revealed binding sites for nuclear hormone receptors within multiple airway DHS [46]. These included the glucocorticoid receptor (GR), binding of which can be induced by dexamethasone treatment, suggesting that *CFTR* may also be subject to hormonal regulation in some cell types.

The mechanism(s) responsible for the marked divergence in *CFTR* expression levels within airway (low) and intestinal (high) epithelial cells [44] are of substantial clinical relevance, since they might provide clues to new therapies based on altering gene expression levels. It is probable that not only are differences in cell-type-selective enhancers relevant, together with the recruitment of divergent activating transcription factors, but that a repressive mechanism modulates basal *CFTR* transcription in the airway. In this context, recent work suggesting that an airway-selective regulatory mechanism may involve a repurposing of the transcription factors FOXA1/A2 and CEBPα, which activate *CFTR* in the intestine to function as repressors in airway cells [58] is of interest.

## 5. Insulator Elements at the *CFTR* Locus

In addition to the DHS identified at the *CFTR* locus that encompass enhancers or tissue-specific regulatory elements, multiple regions of open chromatin are either ubiquitous or present in many cell types [44]. Among these features are DHS flanking the locus on the 5' side, at −79.5 kb and −20.9 kb, and others 3' at +6.8 kb, +15.6 kb, and +48.9 kb (Figure 1). Previous experiments established that the −20.9 kb, +6.8 kb, and +15.6 kb sites marked sequences with enhancer-blocking insulator activity in K562 cell based assays [50,59]. Insulators elements were initially identified based on their ability to prevent inappropriate interactions between enhancers and promoters or to demarcate distinct chromatin domains [60]. These *cis*-regulatory regions are especially important at the *CFTR* locus where the flanking genes, ankyrin repeat, SAM and basic leucine zipper domain containing 1(*ASZ1*) on the 5' side [61] and cortactin binding protein 2 (*CTTNBP2*) on the 3' side [62], have very different expression patterns from the *CFTR* locus. To ensure correct regulation of *CFTR*, it could be advantageous to create an isolated chromatin domain around the locus to prevent inappropriate interactions with regulatory elements of the neighboring genes. Insulator sites are often bound by CCCTC-binding factor (CTCF) and are readily identified genome-wide by ChIP-seq for this factor [63]. CTCF is a highly conserved DNA binding protein with 11 zinc fingers that binds to variations of the CCCTC motif [64]. It was initially identified as an activator and repressor and later recognized as the vertebrate insulator protein [65]. Besides its insulator function, CTCF is involved in many different nuclear functions including intra- and inter-chromosomal interactions [66] leading to its reclassification as a genome architectural protein [67].

The *CFTR* DHS elements at −20.9 kb and +6.8 kb contained binding motifs for CTCF, were occupied by CTCF as measured by ChIP, and their insulator function was shown to be dependent on CTCF [50,59]. Interestingly, although the +15.6 kb site [34] displayed insulator activity, this was independent of CTCF and might involve nuclear hormone receptors [50]. CTCF performs its functions at approximately 60%–70% sites across the genome in concert with the cohesin complex [68,69,70]. The core cohesin complex is composed of several proteins including structural maintenance of chromosomes 1 (SMC1), SMC3, SCC1 (RAD21), and SA1/SA2, which form a ring-like structure to encircle chromatin [71,72]. ChIP-seq data from the ENCODE project confirmed that the DHS at −20.9 kb and +6.8 kb were enriched for CTCF and cohesin components [42]. Also, our ChIP data on the *CFTR* locus identified CTCF/RAD21 occupancy at these two sites and at the ubiquitous DHS at +48.9 kb, within the last intron of the flanking gene [44,50,59]. Additional CTCF/cohesin binding sites were also found at −80.1 kb to the translational start site, which corresponds to the −79.5 kb DHS we reported previously based on Southern blotting [32]. The −80.1 kb element was shown to interact with the *CFTR* promoter [73] and is also in close physical association with a CTCF binding site at +83.7 kb from the 3' end of *CFTR* [42,74]. These data show *CFTR* as a paradigm for the multiple mechanisms of transcriptional regulation that are integrated in the control of a single human gene: at the promoter, at tissue-specific enhancer elements, and at CTCF/cohesin bound sites that may be acting as insulators.

## 6. Higher-Order Organization of the *CFTR* Locus

The data discussed in the previous sections of this review convincingly demonstrate that multiple *cis*-regulatory elements are active at the *CFTR* locus, many of them located at a great distance (>100 kb) from the gene promoter. A probable mechanism for these distal elements to regulate *CFTR* is through the establishment of long-range chromatin loops with the promoter and/or with each other. Chromatin looping between promoters and DHS harboring regulatory elements is observed at many different loci, and is particularly well-studied at the β-globin locus [75,76]. The spatial organization of the genome and higher order chromatin structure, whereby chromatin loops can bring together distinct regulatory elements, is critical for transcription and gene regulation [77,78]. Recent mapping of associations between TSS and regulatory elements revealed that TSS generally interact with regions ~120 kb away and only 7% of observed looping of regulatory elements occurs with the nearest gene [79].

Long-range chromatin interactions can be measured using several different chromosome conformation capture technologies: 3C [80], 4C-seq [81], 5C [82], HiC [83], and ChIA-PET [84]. One of the original methods, 3C, measures associations between a fixed or bait site and many other defined sites across a locus using PCR or quantitative PCR [80]. This technique was used to investigate interactions across the *CFTR* locus in multiple cell types [44,73]. Interactions were detected between the promoter bait and DHS marking *cis*-regulatory elements within introns and flanking the locus, only in cells expressing *CFTR*. No significant associations were observed between the promoter and the same sites in cell types where the locus is silent, suggesting that looping is dependent on the DHS being present and the elements such as enhancers being actively bound by transcription factors [44,55,73]. Furthermore, many of the interactions between different regulatory elements were shown to be reciprocal using multiple bait regions in the 3C experiments [73]. In airway cells, where distinct regulatory elements are utilized, interactions were observed between the *CFTR* promoter, DHS −44 kb and DHS −35 kb, though low or no significant interactions were seen with the intestinal-selective elements such as the enhancer in intron 11 [44,55,74].

An effort to reveal the mechanisms that mediated the formation of these looped chromatin structures at the *CFTR* locus focused on CTCF and the cohesin complex, which are known to be recruited to the gene [44,50,59]. The function(s) of these factors were examined across the 7q31.2 genomic region encompassing the *CFTR* locus using a siRNA-mediated depletion approach. Loss of CTCF had a significant impact on the long-range associations between CTCF/cohesin binding sites flanking the locus, but did not alter the interaction of the promoter with intronic *cis*-elements [74]. In contrast, depletion of RAD21 reduced all the interactions across the locus including those between the promoter, enhancers, and other *cis*-regulatory elements including CTCF/cohesin bound regions, suggesting cohesin was primarily stabilizing the different sets of chromatin loops [74]. These data suggested that CTCF and cohesin performed distinct functions at the *CFTR* locus. Recent genome-wide studies confirmed our observations [85] and provided evidence for a CTCF-independent role for cohesin in enhancer-promoter interactions [86]. Specifically, cohesin depletion caused a decrease in intra-chromosomal interactions, while loss of CTCF increased associations between topologically associated domains (TADs) [85], which are large, mega-based sized domains that under normal conditions show lower interaction frequencies with each other [67]. Hence, at the *CFTR* locus, where CTCF mediates the loops between flanking CTCF/cohesin binding sites, those regions may mark the boundaries of a TAD that isolates the locus for appropriate regulation. Our recent 4C-seq data generated across the *CFTR* locus support this hypothesis (Yang *et al.*, unpublished). The cohesin complex stabilizes interactions between TSS and *cis*-regulatory elements such as enhancers at many loci including the *CFTR* locus. However, despite the changes in the three-dimensional conformation of the locus after depletion of CTCF and/or cohesin, *CFTR* mRNA and protein levels increased [74]. Further analysis revealed that the increase in expression was associated with alterations in the chromatin landscape, transcription factor occupancy, and nuclear positioning of the *CFTR* alleles.

The role of the cohesin complex in stabilizing associations between the *CFTR* promoter and *cis*-regulatory elements/enhancers implicated transcription factors binding to these regions in the looping mechanism. Since the FOXA1/A2 pioneer factors were identified as the pivotal proteins in the transcriptional complex regulating *CFTR* intestinal enhancers [52], their role in the three-dimensional organization of *CFTR* was examined. Combined depletion of both FOXA1 and FOXA2 significantly decreased interactions between the promoter and important intestinal *cis*-regulatory elements located in introns 10 and 11 [52]. Although there was evidence for an indirect role of the FOXA family members in chromatin looping genome-wide at estrogen receptor binding sites [53,84], the *CFTR* data may be the first evidence of a direct role of FOXA1/A2 in chromatin organization. Recent work also identified a role for the chromatin remodeler, chromodomain helicase DNA-binding 6 (CHD6), in regulating *CFTR* expression. CHD6 binds to several *cis*-regulatory elements at the *CFTR* locus, including the DHS at intron 1, and interacts with members of the facilitates chromatin transcription (FACT) complex and other transcription factors such as CDX2 [87]. shRNA-mediated depletion of CHD6 decreased *CFTR* mRNA levels and reduced long-range chromatin looping across the locus, indicating its importance both as an activating factor and a chromatin organizer at the locus. However, in these experiments depletion of CTCF in CF-PAC cells reduced *CFTR* transcript levels in contrast to the increase we observed in Caco2 cells. This suggests there may be some cell specificity in the role of architectural proteins in organizing the active locus or that the relative abundance of *CFTR* transcripts in different cell types may be important.

## 7. Conclusions

In summary, though the *CFTR* promoter is necessary to drive basal levels of gene expression, *cis*-elements located elsewhere in the locus are required to confer tissue specificity and control abundance of the transcript. These elements interact directly with the promoter via chromosome looping and perhaps other mechanisms, and recruit diverse cell-selective transcription factors.

The extensive studies carried out over many years, which are discussed in this review, have provided substantial insights into the temporal and tissue-specific regulation of *CFTR* expression, though our understanding remains incomplete. They also illustrate the critical importance of investigating the locus in differentiated cell types from many lineages, which utilize different transcriptional networks. More recently, the integration of data on *cis*-regulatory elements, their *trans*-interacting factors, and the contribution of higher order chromatin structure have illustrated the important role of chromatin dynamics in the regulatory mechanism. Many of the insights gained from *CFTR* are equally relevant to other disease-associated human genes and coincide with observations from multiple, elegant genome-wide analyses. Future work will identify the specifics of critical cell-type selective transcription factors and regulatory elements in primary cells from the airway, intestinal organoids, sweat gland ducts, and the epididymis, where *CFTR* expression is required for normal epithelial function. Moreover, the ultimate proof for the importance of these *cis*-regulatory elements *in vivo* will require deleting the elements individually and in combination using systems such as CRISPR-Cas9 [88,89] to assess their affect on *CFTR* expression, its chromatin landscape, and higher order organization. Use of the same technology to modify transcription factor binding sites within individual elements may also identify the proteins critical for maintaining appropriate, tissue-specific levels of *CFTR* expression (see many online tools for recent CRISPR applications). Finally, it will be important to incorporate our understanding of post-transcriptional mechanisms of *CFTR* regulation, for example by microRNAs (miRNAs), with knowledge of the critical role of chromatin dynamics at the locus. Though not a focus of this review, *CFTR* is also subject to direct repression via its 3' UTR by several miRs including miR-145 and miR-494 [90,91,92]. *CFTR* may also be indirectly regulated by miR-138, which inhibits SIN3A [93], a transcriptional repressor that regulates the gene and alters its chromatin dynamics [56]. Integrating these diverse regulatory mechanisms may ultimately facilitate approaches to manipulate *CFTR* gene expression for therapeutic benefit.
